# When the Party Goes Wrong: A Case Report of Recurrent Valsalva Retinopathy Treated With Pars Plana Vitrectomy

**DOI:** 10.7759/cureus.50237

**Published:** 2023-12-09

**Authors:** Renato Correia Barbosa, Rui Carvalho

**Affiliations:** 1 Ophthalmology, Hospital Pedro Hispano, Unidade Local de Saúde de Matosinhos, Matosinhos, PRT

**Keywords:** retina surgery, pars plana vitrectomy, laser membranotomy, valsalva retinopathy, preretinal hemorrhage

## Abstract

Valsalva retinopathy is a preretinal hemorrhage caused by a sudden increase in intrathoracic or intra-abdominal pressure, which generally happens after sudden and intense physical effort. This case report describes a case of Valsalva preretinal hemorrhage first treated with laser membranotomy, which subsequently recurred and was retreated with a pars plana vitrectomy.

An 18-year-old male was admitted due to complaints of decreased visual acuity in his right eye for three days. He had been practicing strength training in the gymnasium before the complaints started but denied trauma or other precipitating factors. Fundoscopy revealed a central preretinal hemorrhage, and he underwent laser membranotomy, which successfully released the blood from the sub-hyaloid space into the vitreous cavity. However, the following night, he went to a nightclub party and then returned with the same initial symptoms of decreased visual acuity in his right eye. Fundoscopy revealed a relapse of the hemorrhage, which was now too central for membranotomy. He was proposed for a pars plana vitrectomy, with an aspiration of the blood, which was found to be under the inner limiting membrane. The patient achieved complete functional recovery after two weeks, with visual acuities of 20/20 on his right eye.

Valsalva retinopathy may be treated with a conservative non-interventional approach, but laser membranotomy and surgery may be beneficial in selected cases, promoting faster visual rehabilitation and avoiding potential long-term toxicity effects of the prolonged presence of preretinal blood. Compliance with the postoperative rest period is essential after a laser membranotomy is performed, and failure to do it may result in the recurrence of the hemorrhage.

## Introduction

Preretinal hemorrhages consist of the accumulation of blood under the posterior hyaloid or under the internal limiting membrane (ILM) of the retina. The most common causes include Valsalva retinopathy, Terson syndrome, proliferative diabetic retinopathy, and retinal vascular occlusions [1].

Valsalva retinopathy is a preretinal hemorrhage caused by a sudden increase in intrathoracic or intra-abdominal pressure, which generally occurs in otherwise healthy eyes. The sudden rise in thoracic or abdominal against a closed glottis, called the Valsalva maneuver, reduces the venous return to the heart and the stroke volume, which in consequence increases the venous system pressure. This increased venous pressure, which mainly affects the upper part of the body, due to gravity, results in the increase of the intraocular venous pressure, which in turn leads to the rupture of the superficial retinal capillaries and extravasation of blood in the retina [2,3]. It is often related to activities such as vomiting, coughing, straining for defecation, weightlifting, sexual intercourse, and labor, but any kind of strenuous physical activity may be associated.

The diagnosis of this disease is based on the clinical history and ophthalmological examination. Main complaints usually include a painless loss of visual acuity, associated with a central or paracentral scotoma, which is often unilateral, although in rare cases may be bilateral. The anterior segment examination may show subconjunctival hemorrhages, or be completely innocent. Fundoscopy reveals a preretinal hemorrhage, typically located in the macular area. The blood can be trapped under the ILM, or in the sub-hyaloid space. The amount of blood is variable, and it is usually displaced in a well-circumscribed, round shape [4].

The treatment of this disease may involve conservative management with the observation of spontaneous resolution, which usually occurs within weeks or months. However, in cases of large hemorrhages, neodymium-doped yttrium aluminum garnet (Nd:YAG) laser membranotomy may be performed to disrupt the ILM or posterior hyaloid and allow the drainage of the blood into the inferior vitreous, resulting in a faster resolution [5,6]. In selected cases, a pars plana vitrectomy (PPV) with the removal of the ILM and aspiration of the hemorrhage may be an alternative therapeutic option.

This case report aims to describe a case of a Valsalva retinopathy initially treated with a Nd:YAG membranotomy, which subsequently recurred and underwent a PPV with peeling of the ILM and aspiration of the pre-retinal blood.

## Case presentation

**Figure 1:**
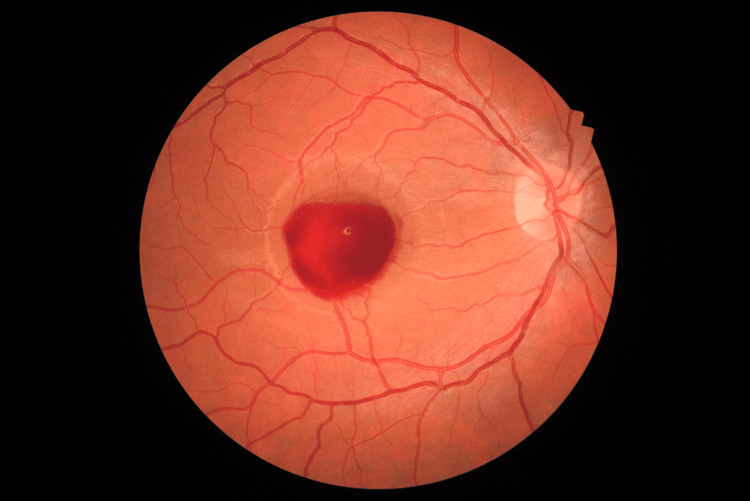
Color fundus photograph showing a central preretinal hemorrhage

An 18-year-old male was admitted to the emergency department due to complaints of decreased central vision in his right eye (OD) for three days. He had no relevant medical or surgical history and took no medication. He was practicing strength training in the gymnasium before the complaints started but denied trauma or other precipitating factors. At the examination, he had a best corrected visual acuity (BCVA) of counting fingers on his OD and 20/20 on his left eye (OS). The pupillary examination was normal, and eye movements were unrestricted. Intraocular pressures (IOPs) were also normal. Slit-lamp examination revealed a normal anterior segment, without anterior chamber reaction. He underwent pharmacological mydriasis, and fundoscopy showed the presence of a central retro-hyaloid hemorrhage (Figure 1), which was confirmed by spectral-domain optical coherence tomography (SD-OCT).

**Figure 2:**
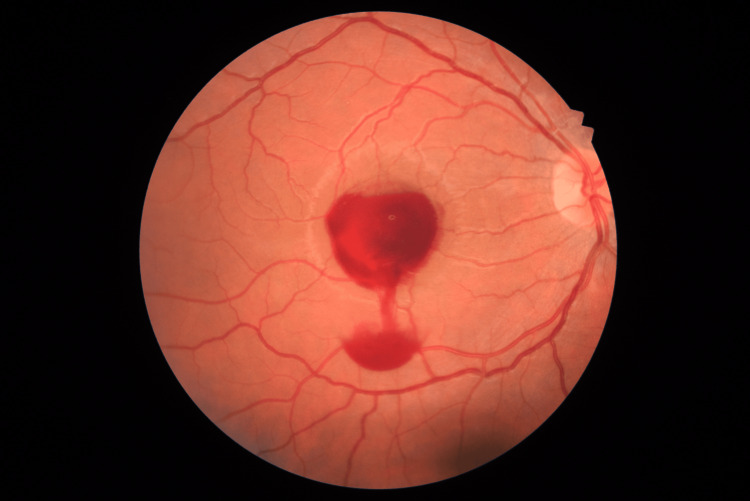
Color fundus photograph of the macula right after the Nd:YAG membranotomy Nd:YAG: neodymium-doped yttrium aluminum garnet.

He underwent an Nd:YAG membranotomy, which was performed without complications and resulted in the immediate release of blood from the sub-hyaloid space to the vitreous cavity (Figure 2).

The patient was then instructed to rest, reinforce hydration, and sleep in an inclined bed position, to further drain the blood by gravity. On the following day, he was re-evaluated and the hemorrhage had partially subsided, with some blood accumulated in the vitreous. The OD BCVA was now 2/20.

**Figure 3:**
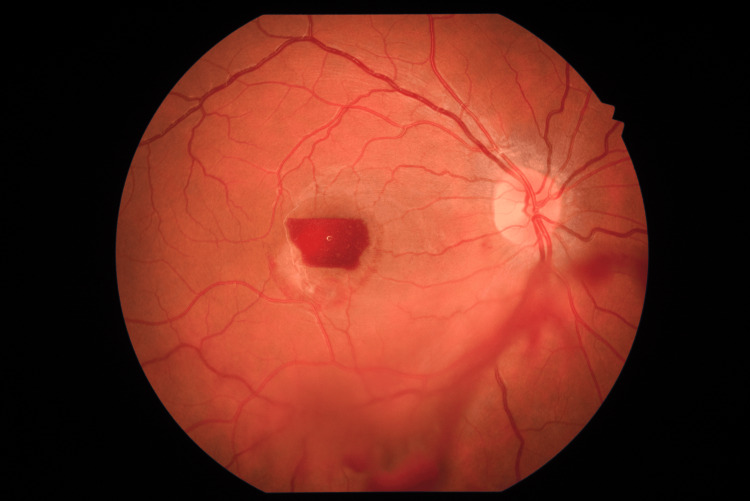
Color fundus photograph of the macula showing relapse of the central preretinal hemorrhage

However, two days after the initial visit, the patient returned to the hospital due to a relapse of the OD visual acuity, which he felt after he went to a party at night, the day before. Fundoscopy now showed a relapse of the hemorrhage with a larger amount of blood in the sub-hyaloid space (Figure 3).

Now the collection was too central to enable another hyaloidectomy, so he was proposed a PPV with the separation of the posterior hyaloid for the aspiration of the hemorrhage.

**Figure 4:**
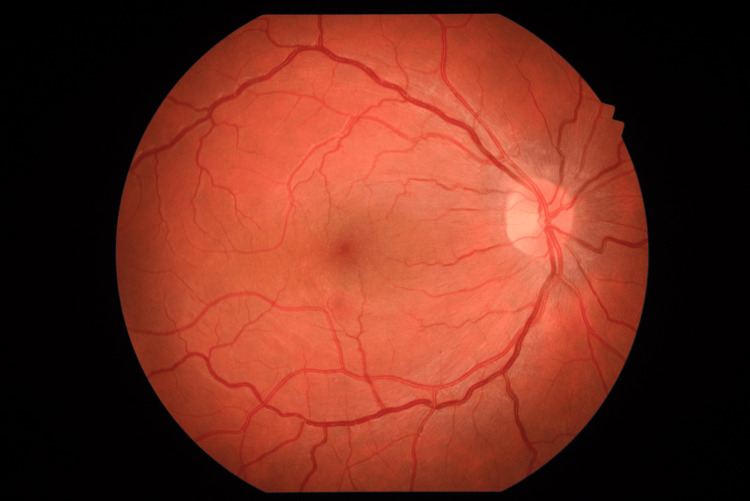
Color fundus photograph showing a clear macula two weeks after the vitrectomy

During the surgery, the posterior hyaloid was detached, and blood was found to be under the inner limiting membrane, which was also peeled. The hemorrhage was then successfully drained, and the posterior segment was left with air. In the postoperative period, normal IOPs were objectified, and a clear macula was visualized (Figure 4).

After seven days, the OD BCVA was already 12/20, and after two weeks, it was 20/20. The patient was instructed to have a normal life but to moderate-severe physical activity, such as weightlifting, in the following months.

## Discussion

Valsalva retinopathy occurs when an increase in systemic venous pressure causes the rupture of superficial retinal capillaries and the extravasation of blood in the preretinal space. The patients are often young and have otherwise healthy eyes. The priority upon suspicion of this identity should be the exclusion of other diseases that may also result in pre-retinal hemorrhages, such as diabetic retinopathy, hypertensive retinopathy, Terson syndrome, Purtscher’s retinopathy, rupture of a retinal macroaneurysm, and inflammatory or infectious diseases of the posterior pole. The differential diagnosis of those pathologies is based on the clinical history and ocular examination. Patients with Valsalva retinopathy are generally healthy, without systemic or ocular risk factors. Slit-lamp examination of the anterior segment is often innocent or shows the presence of subconjunctival hemorrhages. Fundoscopy allows the exclusion of signs characteristic of other diseases such as the presence of microaneurysms and microhemorrhages, cotton wool spots, drusen, retinitis, or other vascular abnormalities [4,7-9].

Laser membranotomy was first described by Fechner in 1980 [10], who used an argon laser. In 1988, Faulborn reported the first Nd:YAG membranotomy [11], and since then, practically all procedures have been carried out with this type of laser, due to its lower risk of retinal damage and subsequent atrophic scars. This treatment modality allows the trapped blood to escape into the vitreous, freeing the fovea from the cover of blood, resulting in a much faster visual recovery. It usually uses a Q-switched Nd:YAG laser in single burst mode, with single shots usually ranging from 2.5 to 10 mJ. The puncture should be placed in the inferior part of the hemorrhage, not directly over the fovea, to facilitate the subsequent gravitational drainage of the blood and to avoid the risk of foveal damage. A substantially thick layer of blood should be present at the aiming site, to shield the retina from the laser. If the procedure is performed correctly, complications are rare, but cases of macular holes, epiretinal membranes, and retinal hemorrhage have been reported [12]. In selected cases, such as large hemorrhages, or those refractory to conservative or laser treatment, PPV may play a role, allowing for the mechanical removal of the preretinal blood after the separation of the posterior hyaloid and the ILM. This technique should be used in select cases, as it is associated with potentially greater risks than Nd:YAG membranotomy, such as cataract formation and increased risk of glaucoma in the long term. On the other hand, it allows for a much faster recovery of the visual function when the laser membranotomy is not effective, or after refractory cases, as the one described in this report. The most important advantage over a conservative approach is that it prevents the toxic effects and the anatomical disruption of the prolonged presence of blood in the fovea, which may lead to irreversible damage to the retina, even after the blood has been completely reabsorbed. Although the toxic effect of blood is inferior in cases of pre-retinal hemorrhage, when compared to intra- or subretinal hemorrhages, it plays a non-negligible role. In addition, the development of an epiretinal membrane is also frequently described when patients undergo conservative therapy, which will often culminate in the need for a PPV in the medium term [12].

Our clinical case presents an unusual sequence of events. Our patient did not respect the recovery period after the procedure and went to a disco party the following night, which resulted in the hemorrhage recurring. After this recurrence, the new blood collection, although quite dense, was too central to allow a new membranotomy to be performed, so vitrectomy was the proposed solution. During the procedure, it was noticed that the blood collection was under the ILM, which was then peeled with subsequent complete aspiration of the subretinal blood. This time, after the surgery, the patient respected the postoperative recovery period, and the final functional results were very satisfying, with a BCVA of 20/20, two weeks after the procedure.

## Conclusions

This case report concluded two main aspects of the treatment of Valsalva retinopathy. Firstly, there can be a recurrence of the pre-retinal hemorrhage after laser membranotomy in cases where the postoperative recovery period is not respected. On the other hand, it showed that in the event of a recurrent pre-retinal hemorrhage, treatment with PPV can be a valid alternative to remove the reaccumulated blood, especially if the collection is large and too central, allowing for a fast recovery of the visual function.
